# Filaricidal properties of *Lantana camara* and *Tamarindus indica* extracts, and Lantadene A from *L*. *camara* against *Onchocerca ochengi* and *Loa loa*

**DOI:** 10.1371/journal.pntd.0006565

**Published:** 2018-06-13

**Authors:** Adela Ngwewondo, Meng Wang, Faustin Pascal T. Manfo, Moses Samje, Jessie N’kam Ganin’s, Emmanuel Ndi, Raymond J. Andersen, Fidelis Cho-Ngwa

**Affiliations:** 1 Department of Biochemistry and Molecular Biology, Faculty of Science, University of Buea, Buea, Cameroon; 2 Institute of Medical Research and Medicinal Plants Studies (IMPM), Yaounde, Cameroon; 3 Department of Chemistry, University of British Colombia, 2036 Main Mall, Vancouver, BC Canada; 4 Department of Biomedical Sciences, Faculty of Health Sciences, University of Bamenda, Bambili, Cameroon; Federal University of Agriculture, NIGERIA

## Abstract

**Background:**

Ivermectin is the only drug currently recommended for the treatment of onchocerciasis, the second leading infectious cause of blindness in the world. This drug kills only the first stage larvae—microfilariae (mf) of *Onchocerca volvulus* and is to be used cautiously in areas where *Loa loa* is prevalent because of severe adverse events observed with coinfected patients.

**Methodology/Principal findings:**

This study investigated the anti-filarial activities of two Cameroonian medicinal plants, *Lantana camara* and *Tamarindus indica* locally used to treat onchocerciasis. Twelve (12) extracts were prepared and tested *in vitro* on the bovine model parasite, *O*. *ochengi* as well as *L*. *loa* mf. Both mf and adult male worm viabilities were assessed by motility scoring, while adult female worm viability was determined biochemically by standard MTT/formazan colorimetry. Cytotoxicity and acute toxicity were determined respectively, in monkey kidney epithelial cells and in BALB/c mice. Pure compounds were isolated by LC/MS using a bio-assay guided strategy. All the extracts showed 100% activity at 500 μg/mL against *O*. *ochengi* adult worms and mf. The highest activity against *O*. *ochengi* was observed with the hexane extract of *L*. *camara* leaves (LCL_hex_), with IC_50_ of 35.1 μg/mL for adult females and 3.8 μg/mL for the mf. Interestingly, this extract was more active against *O*. *ochengi* mf than *L*. *loa* mf. Further studies on the extracts led to the isolation of lantadene A from the methylene chloride extract of *L*. *camara* leaves, with IC_50s_ of 7.85 μg/mL for adult males, 10.38 μg/mL for adult females, 10.84 μg/mL for *O*. *ochengi* mf and 20.13 μg/mL for *L*. *loa* mf.

**Conclusions/Significance:**

We report for the first time the anti-onchocercal activities of these locally consumed medicinal plants and lantadene A, a potential lead for further development as an onchocerciasis cure.

## Author summary

Onchocerciasis is a chronic disease of humans that affects mainly the skin and eyes. It is an insect-borne disease, caused by a nematode worm, *Onchocerca volvulus*. It is a public health problem and an obstacle to socio-economic development in affected communities. There is currently no vaccine, and no adult worm drug to cure the infection. The only recommended drug, ivermectin can give short-term relief by killing the microfilariae of the parasite but cannot always be safely administered in mass drug administration (MDA) in areas where another filarial worm, *Loa loa* is co-endemic. To prevent infection and completely eradicate the disease there is an urgent need for alternative drugs that can kill the adult worms but to a lesser extent *Loa loa*. In the past, medicinal plants have served as good starting points for the development of drugs. In the present study, we determined the filaricidal properties of extracts, chromatographic fractions of *Lantana camara* and *Tamarindus indica* and lantadene A against *Onchocerca ochengi*, the bovine model of the parasite and *L*. *loa*. Interestingly, most of the active extracts were more active against *O*. *ochengi* than on *L*. *loa* mf. No death was recorded following oral administration of the active extracts to BALB/c mice. Lantadene A is a potential lead molecule for the development of a cure for onchocerciasis.

## Introduction

Onchocerciasis (river blindness) is a blinding and debilitating disease caused by the parasitic nematode, *Onchocerca volvulus*. According to estimates of the World Health Organization (WHO) [1], 37 million people are infected, 800,000 visually impaired and 270,000 blinded. Adult worms of *O*. *volvulus* can live for up to 15 years in subcutaneous nodules (onchocercoma) and produce millions of microfilariae (mf) which parasitize skin and eye tissues, resulting in major pathologies such as intense and often unbearable itching, disfiguring dermatitis, atrophy, visual impairment and blindness [2]. The microfilaricide, ivermectin was shown to be safe and effective in the treatment of onchocerciasis and is currently the only recommended drug for control of the disease by a mass drug administration (MDA) strategy [3]. The emergence of animal parasite strains resistant to ivermectin and an abundance of reports of resistance or low response rates of *O*. *volvulus* mf to the drug are worrisome. Additionally, the use of ivermectin in MDA in areas of high *Loa loa* co-endemicity is limited due to severe adverse events (including encephalopathy and death) observed with some coinfected patients [4]. Since ivermectin is only effective against the mf, prolonged annual therapy for at least 10 to 15 years is required to interrupt transmission and clear onchocerciasis from a human population [5]. Therefore, there is the need for a safe and more effective macrofilaricidal drug for the cure of onchocerciasis or an alternative microfilaricide, preferably one that does not kill *L*. *loa* mf. Since onchocerciasis is a neglected tropical disease, such a drug has been difficult to find with the conventional for-profit pharmaceutical company approach, requiring alternative strategies to aid its discovery and development.

One strategy employed has been the exploitation of medicinal plants and other natural materials as alternative medicines or for the identification of novel potential drug leads. It has been shown that medicinal plants play a very important role in the health care needs of rural populations in Africa because they are cheap and readily available locally [6,7]. The majority of drugs active against infectious agents are in fact derived from natural products [8], including ivermectin derived from *Streptomyces avermitilis* [9] and artemisinin from the medicinal plant, *Artemisia annua* [10]. Previous studies have revealed the filaricidal properties of several Cameroonian medicinal plants [11–13]. *Tamarindus indica* lotions and extracts are widely used by indigenes to treat conjunctivitis, dysentery, jaundice, hemorrhoids and onchocerciasis. *Lantana camara* contains principles active against *Mycobacterium tuberculosis* and has been used in the traditional treatment of onchocerciasis in parts of Cameroon [14]. This study thus sought to investigate the filaricidal properties of extracts from *Lantana camara* (Verbenaceae) and *Tamarindus indica* (Leguminosae) against cattle derived *O*. *ochengi*, the closest known relative of *O*. *volvulus* [15], and against *L*. *loa* mf, in order to assess their acclaimed activities and their possible use as sources of new drug leads for onchocerciasis.

## Materials and methods

### Ethics statement

Ethical clearance (No. 2013/11/371/L/CNERSH/SP) and administrative clearance (No. 631–06.14) for blood collection from *L*. *loa* infected humans were obtained from the Cameroon National Ethics Committee and the Ministry of Public Health, respectively. All subjects of age 20–55 granted written and informed consent before any blood for diagnosis or worm preparation was collected.

### Plants and plant extracts

Both *L*. *camara* and *T*. *indica* were collected in January, 2013 based on ethno pharmacological information from Bafoussam and Oshei communities in the West and North West Regions of Cameroon, respectively. Voucher specimens were taken to the Yaoundé herbarium where they were authenticated by Mr. Onana Jean Marie and voucher numbers were assigned to them (*L*. *camara*: 25900 SRF CAM; *T*. *indica*: HNC/42429). The leaves, stem bark and roots of each plant were air dried and ground to fine powder using a grinding mill. Each powder was macerated for 48 hours, sequentially, in hexane, methylene chloride, and methanol. The filtrate was concentrated using a rotary evaporator (BUCHI Rotavapor R-200, Switzerland) and crude extracts weighed and preserved at -20°C for further use.

The percentage yield in extract was calculated using the following formula:

%yield=(Weightofcrudeextractx100)/Weightofdrygroundedplantmaterial

A stock solution of 25 mg/mL was prepared in >99.8% DMSO (Sigma, Germany) and kept at -20°C until tested in biological assays.

### Isolation of *O*. *ochengi* adult worms

Worms were isolated from umbilical areas of infected cattle skin as previously described by Cho-Ngwa *et al*. [11]. Briefly, cattle skin containing palpable nodules obtained from the butchery in Douala Cameroon were washed with soap and rinsed with distilled water. The skin was then sterilized with 70% ethanol after which the nodules were carefully opened and the entire nodular content removed and submerged in 2 mL of complete culture medium (CCM) comprising of RPMI-1640 (SIGMA cat: R0883), supplemented with 25 mM HEPES, 2 g/L sodium bicarbonate, 2 mM L-glutamine, 5% heat inactivated new born calf serum (SIGMA Cat: N4762), 200 units/mL penicillin/ 200 μg/mL streptomycin and 0.25 μg/mL amphotericin B, pH 7.4 in 12-well culture plates. The worms were left in cultures in a HERACELL-CO_2_ incubator (Thermo Fisher, UK) overnight and checked for any contamination before drugs were added.

### Mammalian cells for microfilarial cultures and cytotoxicity studies

Monkey kidney epithelial cells (LLC-MK2) obtained from the American Type Culture Collection (ATCC) were proliferated in 96-well microtitre plates in CCM medium at 37°C in 5% CO_2_ humidified air. At confluency, the cells served as feeder layers for the mf cultures.

### Isolation and culturing of *O*. *ochengi* microfilariae

This was prepared as described by Bianco *et al*., [16] with slight modifications. Briefly, fresh pieces of umbilical cattle skin were obtained from the butchery and washed thoroughly. Few skin snips from different locations of the skin were obtained and incubated in small amounts of culture medium for 15 minutes, after which the emergent mf were qualified and quantified using an inverted microscope and standard atlases for reference [17]. The remainder of a selected piece of skin was shaved, rinsed and sterilized with 70% ethanol and sliced into thin slivers. The slivers were incubated in CCM for 2 hours, and the emergent highly motile *O*. *ochengi* mf were concentrated by centrifugation. The mf were transferred into 96 -well microtitre plates (15 mf /100 μL/ well) already containing fully confluent LLC-MK2 cell layer in 100 μL of CCM and monitored for viability and sterility for 24 hours before addition of test and control compounds.

### Preparation of *Loa loa* microfilariae

Identification of *L*. *loa* mfs was done using standard atlases after staining with giemsa and observing the slide under the microscope [18]. Whole blood was collected in an EDTA tube from patients not receiving treatment and transported immediately to the Laboratory. The mf load was determined with the aid of an inverted microscope after diluting a portion of the blood in RPMI-1640 medium. After this the blood was diluted according to the number of mf present at initial count so as to obtain a total of 15 mf/100 μL/well. After dilution, the mf were distributed in 96-well plate and monitored for 24 hours before addition of test and control compounds.

### Primary screens against *O*. *ochengi* adult worms

Extracts were tested at 500 μg/mL in triplicates in CCM. Auranofin at 10 μM, which had previously shown activity against *O*. *ochengi* adult worms and mf [19] was used as positive control, while negative control wells received the diluent, 2% DMSO only, previously shown to have no effect on parasite viability. The worm cultures with drug were incubated for 168 hours (7 days), at 37°C in 5% CO_2_ atmosphere. On the last day of incubation, the female worms were removed and incubated in 500 μL of 0.5 mg/mL MTT for 30 minutes. Inhibition of formazan formation from MTT directly correlates with worm death. The worms were blotted on absorbent paper and observed visually for blue coloration against a white background. Scores based on activity were assigned, ranging from 100% inhibition of formazan formationcompletely pale yellow worm, 90% inhibition; only one or few spots of blue color seen on worm, 75% inhibition; about 75% of worm remained pale yellow, 50% inhibition; about 50% of worm remained pale yellow, to 25% inhibition; near total blue coloration, to 0% inhibition; for total blue color on worm for inactive compound.

Adult male worm motility was evaluated using an inverted microscope. Scores were attributed to the worms using the following code: Vigorous or normal movement of whole worm, corresponding to 0% inhibition of worm motility; near normal movement of whole worm or 25% inhibition of worm motility; whole body of worm motile but sluggish i.e. 50% inhibition of worm motility; only head or tail of worm moving i.e. 75% inhibition of worm motility; completely immotile worm i.e. 100% inhibition of worm motility.

### Secondary screens against *O*. *ochengi* adult worms

Extracts with 100% activity at primary screens were re-tested as described under primary screens and at serial dilutions of seven concentrations (from 500 to 7.8125 μg/mL), in order to determine the IC_50_ values. The IC_50_ assays were done in triplicates and each experiment repeated for confirmation. The means of all activities at a concentration were calculated and used in the statistical analyses. GraphPad prism version 6.0 (GraphPad Software, CA, USA) was used to generate dose response curves from which the IC_50_ values were obtained.

### Primary screens against *O*. *ochengi* microfilariae

Assays were conducted at 500 μg/mL in duplicates in the 96-well microtitre plates. The positive control drug was amorcazine at 30 μM and negative control was the diluent (DMSO). The mf were incubated with drug for 120 hours in a total of 200 μL of medium. Mf viability was assessed by microscopy once every day and motility inhibition scores were recorded as: 100% (immotile), 75% (only head or tail shaking), 50% (sluggish), 25% (almost vigorous motility), 0% (vigorous motility as with negative control). The day 5 data were used in determining drug activity. Motility inhibition correlates to drug activity.

### Secondary screens against *O*. *ochengi* microfilariae

Extracts showing 100% activity in the primary screens were re-tested as described under primary screens at 8 serial dilutions (from 500–3.9 μg/mL) to determine the IC_50_ values. All assays were repeated at least once. The selectivity index (SI) of each extract was calculated as the ratio of the IC_50_ of the extract on mammalian cell (termed CC_50_) to the IC_50_ on parasites.

### Screens against *Loa loa* microfilariae

All the extracts were screened against *L*. *loa* mf and the IC_50_ values determined. This was done at serial dilutions of eight concentrations (500–3.9 μg/mL), and according to the protocol used to screen extracts against *O*. *ochengi* mf. All assays were repeated at least once for confirmation of results.

### Statistical analysis

Data were analyzed using GraphPad prism 6. The statistical significance of differences in means between the effects of extracts at various concentrations on parasites were determined by one-way analysis of variance (ANOVA), followed by Newman-Keuls multiple comparison tests. A value of *p* < 0.05 was considered significant.

### Bioassay guided fractionation

**Fig 1:**
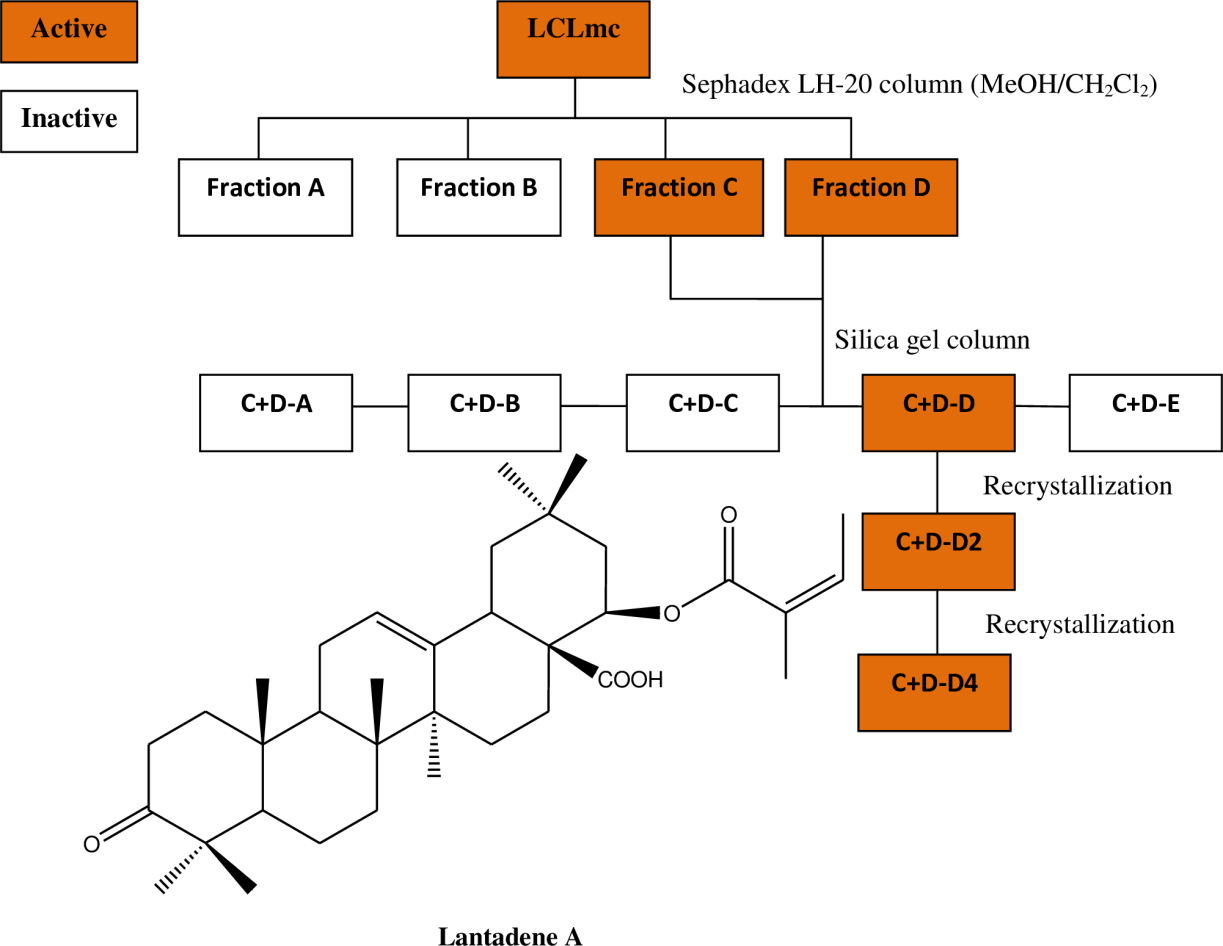
Scheme showing the bioassay guided fractionation of the methylene chloride extract of *Lantana camara* leaves (LCL _mc_) and the structure of lantadene A contained in fraction C+D-D4.

Active extracts were chromatographed on a Sephadex LH-20 column using 4:1 MeOH/CH_2_Cl_2_ as eluent to give fractions A-E which were screened in quadruplicates at 50 μg/mL on all the developmental stages of *O*. *ochengi* and on *L*. *loa* mf. Fractions C and D were combined and further fractionated on a silica gel column with a gradient from 100% hexane to 100% EtOAc to give sub-fractions A-J and screened at the same concentration. Fraction D was recrystallized twice in MeOH to obtain needle-shaped crystals. The crystals were identified as lantadene A by analysis of their NMR and MS data (Fig 1).

Lantadene A: Colorless crystal (4.0 mg); ^1^H NMR (600 MHz, CDCl_3_) *δ* 5.97 (m, 1H), 5.35 (m, 1H), 5.06 (m, 1H), 3.02 (dm, *J* = 13.9 Hz, 1H), 2.53 (m, 1H), 2.35 (dm, *J* = 14.2 Hz, 1H), 1.99 (m, 1H), 1.95 (dm,*J* = 6.5 Hz, 3H), 1.90 (m, 1H), 1.87 (dm, *J* = 13.5 Hz, 2H), 1.79 (br. d, *J* = 14.1 Hz, 2H), 1.74 (m, 1H), 1.73 (br. d, *J* = 5.6 Hz, 3H), 1.65 (m, 1H),1.49 (m, 1H), 1.46 (m, 2H), 1.39 (dm, *J* = 6.7 Hz, 2H), 1.28 (m, 1H), 1.27 (m, 2H), 1.26 (m, 2H), 1.15 (br. s, 3H), 1.07 (br. s, 3H), 1.03 (br. s, 3H), 1.02 (br. s, 3H), 0.98 (br. s, 3H), 0.87 (br. s, 3H), 0.80 (br. d, *J* = 5.7 Hz, 3H); ^13^C NMR(150 MHz, CDCl_3_) *δ*217.9, 179.7, 166.4, 143.3, 139.3, 127.8, 122.7, 76.0, 55.5, 50.8, 47.6, 47.1, 46.1, 42.2, 39.4, 39.3, 38.6, 37.9, 37.0, 34.3, 33.9, 32.4, 30.2, 27.8, 26.7, 26.3, 26.0, 24.4, 23.7, 21.7, 20.8, 19.7, 17.1, 15.9, 15.3; HRESIMS [M+Na]^+^m/z 575.3708 (calcd for C_35_H_52_O_5_Na, 575.3712).

### Screening of lantadene A against the parasites

Lantadene A was first tested at 50 μg/mL in the primary screens against the males, females and mf of *O*. *ochengi* and *L*. *loa* mf. Thereafter, it was tested in secondary screens from 40 μg/mL—0.31 μg/mL for adult worms, 40 μg/mL—0.16 μg/mL for *O*. *ochengi* mf and 40 μg/mL– 10 μg/mL for *Loa loa* mf in order to determine its IC_50_ values, using the same assays described for the extracts.

### Cytotoxicity studies

Cytotoxicity of extracts with anti-*Onchocerca* activities was assessed on LLC-MK2 cells, microscopically, on day 5 of the mf assay. Living cells were flattened out and attached to the culture plate, while dead cells were rounded up and detached from the plate. The IC_50_ values for these cells were estimated from the morphological deformation data.

### Acute toxicity studies

This test was conducted in accordance with the OECD guideline for testing of chemicals [20] and the animal protocol IACUC No UBAP2014-001 was approved by the Animal Care and Use Committee of the Faculty of Science, University of Buea. The three most active extracts (LCL_mc,_ TIL_mc_ and LCL_hex_) were tested for acute toxicity in BALB/c mice.

Thirty-two animals of approximately 21.67g body weight each were divided into 4 groups (each group consisting of 1 subgroup of 4 males and 1 subgroup of 4 females). Each of the 3 treatment groups received one of the extracts at a limit dose of 2000 mg/kg body weight, administered orally in a maximum volume of 280 μL of corn oil per animal; while the control group received the diluent only. The animals were observed for any changes in physical activity, food intake, water intake, stool sample, loss of fur, sensitivity to sound, sensitivity to pain, motility and mortality, every day for 14 days.

### Phytochemical analyses

The phytochemical properties of the active extracts were determined using standard procedures: Mayer and freshly prepared Dragendoff’s reagents for alkaloïds, Liebermann-Buchard test for triterpenoids and sterols, FeCl_3_ and K_3_Fe[(CN)_6_] for phenols, Shinoda’s test for flavonoids, frothing test for saponins [21].

## Results

**Fig 2:**
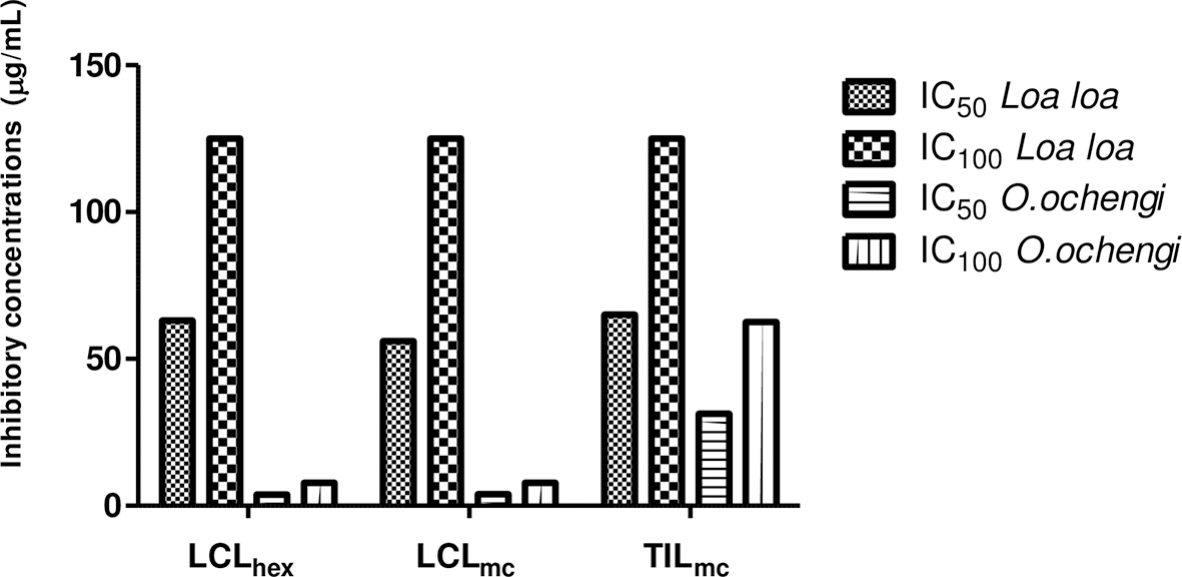
Graphical representation of IC_50_ and IC_100_ of active extracts against microfilariae of *L*. *loa* compared to the values for *O*. *ochengi*. (TIL_mc_: methylene chloride extract from *Tamarindus indica* leaves; LCL_hex_: hexane extract from *Lantana camara* leaves; LCL_mc_: methylene chloride extract from *Lantana camara* leaves).

The twelve (12) crude extracts obtained from the 2 plants, *L*. *camara* and *T*. *indica*, were first tested at 500 μg/mL in primary screens against *O*. *ochengi* worm stages. All of the extracts showed 100% activity against adult worms and mf. The extracts were further screened at various concentrations on adult worms and mf in order to determine their IC_50_s. The hexane and methylene chloride extracts of *L*. *camara* leaves (LCL_hex_ and LCL_mc_, respectively) were the most active against adult male worms with IC_50_s of 7.3 and 7.8 μg/mL, and *O*. *ochengi* mf with IC_50_s of 3.8 and 3.9 μg/mL, respectively. Moreover, LCL_hex_ and methylene chloride extract of *T*. *indica* leaves (TIL_mc_) were the most active extracts against female worms with IC_50_s of 35.1 μg/mL and 62.5 μg/mL, respectively. Seven of the twelve extracts had lower activities (higher IC_50_s) against *L*. *loa* mf than *O*. *ochengi* mf (Table 1). LCL_hex_, LCL_mc_, and TIL_mc_ had IC_50_s of 62.5 μg/mL, 55.6 μg/mL and 64.5 μg/mL, for *L*. *loa* respectively. LCL_hex_ and LCL_mc_ had IC_50_ values for *L*.*loa* 16.4 and 14.3 times higher than that for *O*. *ochengi* mf, respectively (Fig 2).

**Table 1:** Percentage yields and IC_50_s of the extracts in secondary screens for the male, female and microfilariae (mf) of *Onchocerca ochengi* and the mf of *Loa loa*.

Plant part/ solvent for extraction	Extract code	Extraction Yield	IC_50_ (μg/mL) for % inhibition of *O*. *ochengi* male worm motility	IC_50_ (μg/mL) for % inhibition of formazan formation by *O*. *ochengi* female worm	IC_50_ (μg/mL) for % inhibition of *O*. *ochengi* mf motility	IC_50_ (μg/mL) for % inhibition of *L*. *loa* mf motility
*Tamarindus indica leaves/ hexane*	TIL_hex_	3.0	18.3	294.2	62.5	7.8
*Tamarindus indica leaves/ methylene chloride*	TIL_mc_	3.6	250	62.5	31.3	64.5
*Tamarindus indica* roots/ *hexane*	TIR_hex_	2.7	37.9	70.2	62.5	64.5
*Tamarindus indica roots/ methanol*	TIR_met_	6.5	45.4	121.2	385.2	250
*Tamarindus indica stem bark/ hexane*	TIB_hex_	1.5	112.4	178.8	62.5	88.4
*Tamarindus indica stem bark/ methylene chloride*	TIB_mc_	2.2	112.4	102.8	62.5	93.4
*Lantana camara leaves/ hexane*	LCL_hex_	1.2	7.3	35.1	3.8	62.5
*Lantana camara leaves/ methylene chloride*	LCL_mc_	3.3	7.8	125	3.9	55.6
*Lantana camara leaves/ methanol*	LCL_met_	1.1	33.2	111.2	7.3	93.4
*Lantana camara stem bark/ methylene chloride*	LCB_mc_	3.5	9.5	176.8	56.1	125
*Lantana camara stem bark/ methanol*	LCB_met_	5	80.11	222.5	177.6	95.1
*Lantana camara root / hexane*	LCR_hex_	3	58.8	222.5	257.9	257.9

Comparing the mean activities of the different extracts tested against males, females and microfilariae of *O*. *ochengi* and *L*. *loa*, we observed a dose dependent effect from 500–3.90625 μg/mL. At a fixed extract concentration (250–7.8125 μg/mL) there were significant differences (*p* < 0.05) between the different types of extracts. For the same extract type at a fixed concentration there was a difference in the response of males, females and microfilariae of *O*. *ochengi* and *L*. *loa* (Table 2, 3, 4 and 5).

**Table 2:** Comparison of the effect of different extracts and concentrations on the mean activity of *O*. *ochengi* females.

Concentration μg/mL	TIL _hex_	TIL _mc_	TIR _hex_	TIR _met_	TIB _hex_	TIB _mc_	LCL _hex_	LCL _mc_	LCL _met_	LCB _mc_	LCB _met_	LCR _hex_
**500**	100± 0^a^	100± 0^a^	100± 0^a^	100± 0^a^	100± 0^a^	100± 0^a^	100± 0^a^	100± 0^a^	100± 0^a^	100± 0^a^	100± 0^a^	100± 0^a^
**250**	25±10.21^d^	100± 0^a^	100± 0^a^	100± 0^a^	100± 0^a^	100± 0^a^	100± 0^a^	100± 0^a^	100± 0^a^	75±10.21^b^	50±10.21^c^	50±10.21^c^
**125**	25±0^d^	100± 0^a^	75±7.21^b^	75±14.43^b^	0±0^e^	100± 0^a^	100± 0^a^	50±14.43^c^	50±0^c^	25±0^d^	25±0^d^	25±0^d^
**62.5**	0±0^d^	50±0^b^	50±7.21^b^	0±0^d^	0±0^d^	0±0^d^	75±14.43^a^	0±0^d^	25±0^c^	0±0^d^	0±0^d^	0±0^d^
**31.25**	0±0^b^	0±0^b^	0±0^b^	0±0^b^	0±0^b^	0±0^b^	50±0^a^	0±0^b^	0±0^b^	0±0^b^	0±0^b^	0±0^b^
**15.63**	0±0^a^	0±0^a^	0±0^a^	0±0^a^	0±0^a^	0±0^a^	0±0^a^	0±0^a^	0±0^a^	0±0^a^	0±0^a^	0±0^a^
**7.81**	0±0^a^	0±0^a^	0±0^a^	0±0^a^	0±0^a^	0±0^a^	0±0^a^	0±0^a^	0±0^a^	0±0^a^	0±0^a^	0±0^a^
**3.91**	0±0^a^	0±0^a^	0±0^a^	0±0^a^	0±0^a^	0±0^a^	0±0^a^	0±0^a^	0±0^a^	0±0^a^	0±0^a^	0±0^a^

^a,b,c,d,e^: Mean ± SEM values with the same letter for the different extracts are not significantly different at *p* < 0.05 for any given concentration.

Mean ± SEM values with different letters for the different extracts are significantly different at *p* < 0.05 for any given concentration

**Table 3:** Comparison of the effect of different extracts and concentrations on the mean activity of *O*. *ochengi* males.

Concentration (μg/mL)	TIL _hex_	TIL _mc_	TIR _hex_	TIR _met_	TIB _hex_	TIB _mc_	LCL _hex_	LCL _mc_	LCL _met_	LCB _mc_	LCB _met_	LCR _hex_
**500**	100± 0^a^	100± 0^a^	100± 0^a^	100± 0^a^	100± 0^a^	100± 0^a^	100± 0^a^	100± 0^a^	100± 0^a^	100± 0^a^	100± 0^a^	100± 0^a^
**250**	100±0^a^	50±0^b^	100±0^a^	100±0^a^	100±0^a^	100±0^a^	100±0^a^	100±0^a^	100±0^a^	100±0^a^	100±0^a^	100±0^a^
**125**	100±0^a^	0±0^c^	100±0^a^	100±0^a^	93.83±4.17^a^	93.83±4.17^a^	100±0^a^	100±0^a^	100±0^a^	100±0^a^	53.33±5.89^b^	62.5±7.21^b^
**62.5**	91.6±8.4^a^	0±0^d^	75±14.43^a^	73.4±24.5^a^	0±0^d^	0±0^d^	100±0^a^	100±0^a^	62.5±12.5 ^a^^b^	75±14.43^a^	37.5±7.217^c^	58.33±6.25^b^
**31.25**	63.89±12.11^b^	0±0^d^	50±20.41^b^^c^	41.67±5.89^c^	0±0^d^	0±0^d^	75±15.73^b^	100±0^a^	50±7.217^b^^c^	75±17.68^b^	33.3±5.893^c^	33.3±13.16^c^
**15.63**	46.8±3.2^c^	0±0^e^	0±0^e^	0±0^e^	0±0^e^	0±0^e^	75±11.97^b^	100±0^a^	25±0^d^	66.7±15.59^b^	0±0^e^	25±0^d^
**7.81**	0±0^b^	0±0^b^	0±0^b^	0±0^b^	0±0^b^	0±0^b^	0±0^b^	50±0^a^	0±0^b^	0±0^b^	0±0^b^	0±0^b^
**3.91**	0±0^a^	0±0^a^	0±0^a^	0±0^a^	0±0^a^	0±0^a^	0±0^a^	0±0^a^	0±0^a^	0±0^a^	0±0^a^	0±0^a^

^a,b,c,d,e^: Mean ± SEM values with the same letter for the different extracts are not significantly different at *p* < 0.05 for any given concentration.

Mean ± SEM values with different letters for the different extracts are significantly different at *p* < 0.05 for any given concentration

**Table 4:** Comparison of the effect of different extracts and concentrations on the mean activity of *O*. *ochengi* microfilariae.

Concentration (μg/mL)	TIL _hex_	TIL _mc_	TIR _hex_	TIR _met_	TIB _hex_	TIB _mc_	LCL _hex_	LCL _mc_	LCL _met_	LCB _mc_	LCB _met_	LCR _hex_
**500**	100± 0^a^	100± 0^a^	100± 0^a^	100± 0^a^	100± 0^a^	100± 0^a^	100± 0^a^	100± 0^a^	100± 0^a^	100± 0^a^	100± 0^a^	100± 0^a^
**250**	100± 0^a^	100± 0^a^	100± 0^a^	0±0^e^	100± 0^a^	100± 0^a^	100± 0^a^	100± 0^a^	100± 0^a^	75±0^b^	50±0^c^	25±0^d^
**125**	100± 0^a^	100± 0^a^	100± 0^a^	0±0^d^	100± 0^a^	100± 0^a^	100± 0^a^	100± 0^a^	100± 0^a^	75±0^b^	50±0^c^	0±0^d^
**62.5**	50±0^b^	100± 0^a^	50±0^b^	0±0^c^	50±0^b^	50±0^b^	100± 0^a^	100± 0^a^	100± 0^a^	50±0^b^	0±0^c^	0±0^c^
**31.25**	0±0^d^	50±0^c^	0±0^d^	0±0^d^	0±0^d^	0±0^d^	100± 0^a^	100± 0^a^	75±0^b^	50±0^c^	0±0^d^	0±0^d^
**15.63**	0±0^c^	0±0^c^	0±0^c^	0±0^c^	0±0^c^	0±0^c^	100± 0^a^	100± 0^a^	75±0^b^	0±0^c^	0±0^c^	0±0^c^
**7.81**	0±0^b^	0±0^b^	0±0^b^	0±0^b^	0±0^b^	0±0^b^	100± 0^a^	100± 0^a^	0±0^b^	0±0^b^	0±0^b^	0±0^b^
**3.91**	0±0^c^	0±0^c^	0±0^c^	0±0^c^	0±0^c^	0±0^c^	75±0^a^	50±0^b^	0±0^c^	0±0^c^	0±0^c^	0±0^c^

^a,b,c,d,e^: Mean ± SEM values with the same letter for the different extracts are not significantly different at *p* < 0.05 for any given concentration.

Mean ± SEM values with different letters for the different extracts are significantly different at *p* < 0.05 for any given concentration

**Table 5:** Comparison of the effect of different extracts and concentrations on the mean activity of *Loa loa* microfilariae.

Concentration (μg/mL)	TIL _hex_	TIL _mc_	TIR _hex_	TIR _met_	TIB _hex_	TIB _mc_	LCL _hex_	LCL _mc_	LCL _met_	LCB _mc_	LCB_met_	LCR_hex_
**500**	100± 0^a^	100± 0^a^	100± 0^a^	100± 0^a^	100± 0^a^	100± 0^a^	100± 0^a^	100± 0^a^	100± 0^a^	100± 0^a^	100± 0^a^	100± 0^a^
**250**	100±0^a^	100±0^a^	100±0^a^	50±0^c^	100±0^a^	100±0^a^	100±0^a^	100±0^a^	100±0^a^	100±0^a^	75±0^b^	25±0^d^
**125**	100±0^a^	100±0^a^	100±0^a^	0±0^d^	75±0^b^	100±0^a^	100±0^a^	100±0^a^	100±0^a^	50±0^c^	75±0^b^	0±0^d^
**62.5**	100±0^a^	25±0^c^	25±0^c^	0±0^d^	25±0^c^	0±0^d^	50±0^b^	50±0^b^	0±0	0±0^d^	25±0^c^	0±0^d^
**31.25**	100± 0^a^	0±0^c^	0±0^c^	0±0^c^	0±0^c^	0±0^c^	0±0^c^	25±0^b^	0±0^c^	0±0^c^	0±0^c^	0±0^c^
**15.63**	100± 0^a^	0±0^b^	0±0^b^	0±0^b^	0±0^b^	0±0^b^	0±0^b^	0±0^b^	0±0^b^	0±0^b^	0±0^b^	0±0^b^
**7.81**	50±0^a^	0±0^b^	0±0^b^	0±0^b^	0±0^b^	0±0^b^	0±0^b^	0±0^b^	0±0^b^	0±0^b^	0±0^b^	0±0^b^
**3.91**	0±0^a^	0±0^a^	0±0^a^	0±0^a^	0±0^a^	0±0^a^	0±0^a^	0±0^a^	0±0^a^	0±0^a^	0±0^a^	0±0^a^

^a,b,c,d,e^: Mean ± SEM values with the same letter for the different extracts are not significantly different at *p* < 0.05 for any given concentration.

Mean ± SEM values with different letters for the different extracts are significantly different at *p* < 0.05 for any given concentration

The drug concentrations inducing cytotoxicity in 50% of cells (CC_50_) were 46.4 μg/mL, 7.8 μg/mL and 7.8 μg/mL for TIL_mc_, LCL_hex_ and LCL_mc_, respectively. Thus, the selectivity index (SI) values of the extracts for adult worms and mf ranged from 0.12–2.07 (Table 6). In general, the cytotoxicity assay demonstrated that about 57% of the 12 extracts had SI values below 1, a clear indication of cytotoxic tendencies for the crude preparations.

**Table 6:** Cytotoxic concentrations (CC_50_) and selectivity indices (SI) of the extracts on the parasites.

Extract code	CC_50_(μg/mL) on Monkey kidney epithelial cells	Selectivity index (SI) of male *O*. *ochengi* worms	Selectivity index (SI) of female *O*. *ochengi* worms	Selectivity index (SI) of *O*. *ochengi* mf	Selectivity index (SI) of *Loa loa* mf
TIL_hex_	62.5	3.41	0.21	1.0	8.0
TIL_mc_	46.4	0.19	0.74	1.48	0.72
TIR_hex_	62.5	1.65	0.89	1.0	0.97
TIB_hex_	125	1.11	0.70	2.0	1.41
TIB_mc_	62.5	0.56	0.61	1.0	0.67
LCL_hex_	7.8	1.07	0.22	2.07	0.12
LCL_mc_	7.8	0.99	0.62	2.0	0.14
LCL_met_	7.8	0.23	0.07	1.07	0.08
LCB_mc_	31.3	3.29	0.18	0.56	0.25
LCB_met_	125	1.56	0.56	0.70	1.32
LCR_hex_	250	4.25	1.12	0.97	0.97

LCL_hex_, TIL_mc_ and LCL_mc_ were selected for acute toxicity studies in BALB/c mice at a limit dose of 2,000 mg/kg body weight. No sign of acute toxicity was noticed in BALB/c mice. The average weights of the mice increased from 21.67g pre-treatment to 26.67g post-treatment. No change was observed in the physical appearance of the animals throughout the 14-day study period.

Phytochemical screening revealed different classes of secondary metabolites present in the three most active extracts (Table 7).

**Table 7:** Phytochemical analysis for TIL_mc_, LCL_hex_, and LCL_mc_ extracts.

Class/Extract	Alkaloids	Flavonoids	Sterols	Triterpenoids	Saponins	Phenols
LCL_mc_	**+**	**-**	**++**	**++**	**-**	**++**
LCL_hex_	**++**	**++**	**++**	**++**	**-**	**++**
TIL_mc_	**-**	**++**	**-**	**+**	**-**	**++**

-: Absent; +: Present, ++: Abundant

Further fractionation of the active extracts, LCL_hex_, LCL_mc_ and TIL_mc_ yielded sub-fractions that were each screened on *O*. *ochengi* mf and adults at 50 μg/mL. The 6 fractions from LCL_hex_ were inactive against the adult worms and mf, while 5 fractions from TIL_mc_ showed moderate activity against adult male and female worms, although inactive against them. Five fractions were obtained from LCL_mc_ and marked activity was observed with two of the fractions (C and D) at 50 μg/mL against adult worms and mf. Combining these two fractions and fractionating further, 11 sub-fractions were obtained. When tested at 50 μg/mL against the male, female and mf of *O*. *ochengi*, 6 sub-fractions [C+D (C, D, D_2,_ F, G, H)] were very active, 2 sub-fractions [C+D (E, I)] moderately active, while the others [C+D (A, B, J)] were inactive. From the above active fractions, the sub-fraction C+D-D_4_ was obtained, which showed 100% activity at 50 μg/mL (Table 8).

**Table 8:** Activities of fractions and sub-fractions from LCL_hex_, LCL_mc_ and TIL_mc_ extracts and lantadene A against *O*. *ochengi* adult worms and larvae.

SN	Fraction	Quantity (mg)	%Activity at 50 μg/mL against *O*. *ochengi* adult male	%Activity of fractions at 50 μg/mL against *O*. *ochengi* adult female	%Activity of fractions at 50 μg/mL against *O*. *ochengi* microflariae	Cytotoxicity at 50 μg/mL
1	6P7-LCL_hex_-A	2.0	0	0	0	No
2	6P7-LCL_hex_-B	2.5	0	0	0	No
3	6P7-LCL_hex_-C	1.6	0	0	0	No
4	6P7-LCL_hex_-D	2.1	100	25.2	0	No
5	6P7-LCL_hex_-E	1.2	6.9	0	0	No
6	6P7-LCL_hex_-F	1.2	10	0	0	No
7	LCL_mc_-A	1.4	53	0	0	No
8	LCL_mc_-B	1.0	67.5	31.3	0	No
9	LCL_mc_-C	1.6	100	87.5	100	Yes
10	LCL_mc_-D	0.8	100	100	100	Yes
11	LCL_mc_-E	0.9	0	0	100	Yes
12	TIL_mc_-A	1.2	0	67.5	0	No
13	TIL_mc_-B	4.0	0	31.3	25	No
14	TIL_mc_-C	0.8	11.3	63.3	0	No
15	TIL_mc_-D	1.2	56.3	25	25	No
16	TIL_mc_-E	1.6	0	12.5	50	No
Combinations of Active Fractions LCL_mc_ C+D and further fractionation						
17	C+D- A	1.5	50	8.9	0	No
18	C+D- B	1.7	10	15.2	0	No
19	C+D-C	3.4	100	100	100	Yes
20	C+D -D	2.2	100	100	100	Yes
21	C+D -D_2_	1.1	100	100	100	Yes
22	C+D -E	2.9	50	75.2	75	Yes
23	C+D—F	2.0	100	87.7	100	Yes
24	C+D—G	1.8	50	87.7	50	No
25	C+D—H	2.8	100	100	100	Yes
26	C+D—I	1.8	50	77.7	25	No
27	C+D—J	2.2	50	43.9	25	No

**Fig 3:**
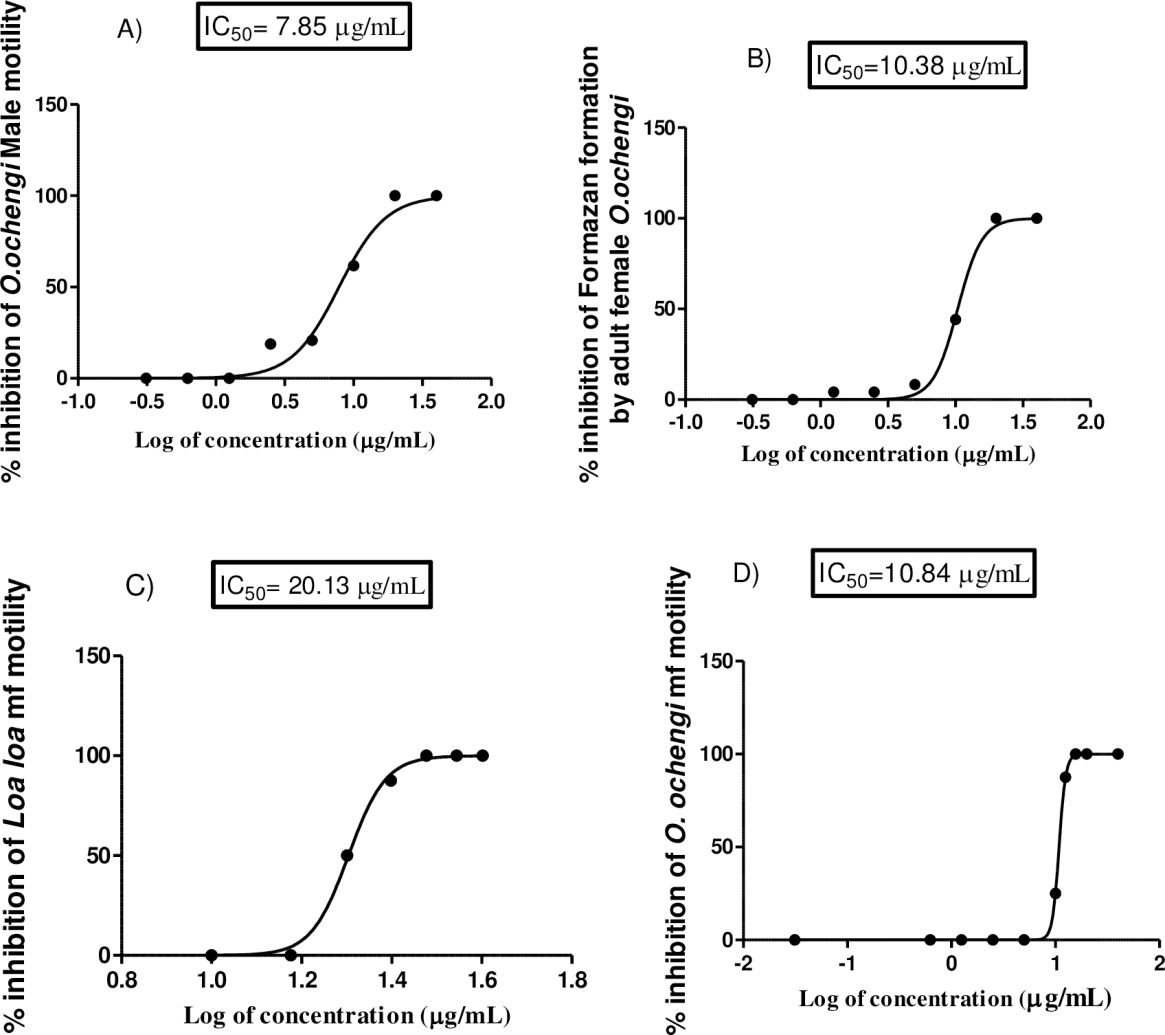
Dose dependent relationships of lantadene A to parasite viability: **A)** % inhibition of motility of *O*. *ochengi* male worms. **B)** % inhibition of formazan by *O*. *ochengi* females. **C)** % inhibition of motility of *Loa loa* microfilariae (mf). **D)** % inhibition of motility of *O*. *ochengi* mf.

Further analysis by liquid chromatography/mass spectrometry (LC/MS) and recrystallisation of combined fractions C+D-D_4_ enabled isolation of the major compound, lantadene A (Fig 1). This pure compound showed 100% activity against *O*. *ochengi* and *L*. *loa* mf at 50 μg/mL in the primary screens; and for the secondary screens, IC_100_ of 20 μg/mL for the adult worms and IC_50s_ of as low as 7.85 μg/mL for the adult male worms; while the IC_100_ and IC_50_ for *L*. *loa* mf were 30 μg/mL and 20.13 μg/mL, respectively (Fig 3).

## Discussion

In this study, we investigated the *in vitro* filaricidal activities of extracts of *L*. *camara* and *T*. *indica*; carried out a bioassay guided fractionation for identification of new drug leads for onchocerciasis and then isolated and determined the filaricidal properties of lantadene A from *L*. *camara* for the first time. Dose-dependent activity relationships were observed with the twelve extracts with IC_50s_ ranging from 385.2 down to 3.8 μg/mL (Table 1). This indicates high anti-onchocerca properties of the plant extracts. The anti-filarial properties of lantadene A, which completely killed the parasites at 20 μg/mL were deemed encouraging, necessitating further studies on the compound.

In the secondary screens, the *L*. *camara* hexane extract (LCL_hex_) and the methylene chloride extract (LCL_mc_) showed significant differences against male worms and *O*. *ochengi* mf when compared to all the extracts. For the adult female worms, significant differences were observed with LCL_hex_ and TIL_mc_ as compared to the other extracts. These differences of a particular extract tested at a single concentration acting differently on the different parasites might be due to the differences in proteins being expressed at the different stages or species. Also the differences observed when testing different extracts on one parasite shows the difference in composition of the extracts. Overall, higher activities were observed with the non-polar extracts than with the polar ones, corroborating previous findings that showed non-polar compounds, including essential oils to be nematicidal [22,23]. It is therefore suggested that, traditional healers find a way of reducing the polarity of their usual aqueous solvents in preparing the corresponding herbal medicines. But this must be confirmed in any clinical trials. The reduction in polarity could be by way of addition of suitable edible oils to the extracting media.

After confirming the filaricidal activity of the extracts, it was deemed necessary to investigate and obtain preliminary data on their safety. About 57% of the extracts tested were more toxic to the monkey kidney epithelial cells than to the worms as reflected in their SI < 1, probably due to complexity of the extracts, although none of the mice died after administration of the selected extracts at limit dose. These results underscore the importance of carrying out full toxicity and dosage studies on traditional medicines, which may generate new problems after the patient might have been cured of the original problem.

Serious adverse events (SAE) associated with incidental killing of *L*. *loa* mf in blood during treatment of patients coinfected with *O*. *volvulus* and *L*. *loa* have been reported [24], suggesting a need for drugs that will selectively kill *O*. *volvulus* without affecting or only moderately affecting *L*. *loa* mf. Interestingly, the most active extracts against *O*. *ochengi* adult males and mf (LCL_hex_ and LCL_mc_) were less active for *L*. *loa* mf (Fig 2). This indicates that these extracts could be potential sources of such selective anti-*Onchocerca* drugs.

To identify novel leads for the development of new drugs for onchocerciasis, bio-assay guided fractionations of the extracts were carried out. Fractions from LCL_hex_ and TIL_mc_ each showed no activity and moderate activity against the parasites respectively, although the whole extract itself was highly active. This implies that active principles from these extracts may be unstable, unable to withstand the fractionation process; or may be acting in synergy to provide the anti-parasitic activity, or even might have been over retarded in the column or missed out in the chromatographic process. Two fractions (C+D) from LCL_mc_ showed marked activity against all the developmental stages of *O*. *ochengi* and *L*. *loa* mf when tested at 50 μg/mL. These yielded the compound lantadene A. The compound had previously been isolated from *L*. *camara* [25]. It is a pentacyclic triperpenoid, with molecular weight 552.78, is only sparingly soluble in water and crystallizes in methanol. It was shown to be active against tumors, *Leishmania* and soil nematodes [26,27]. At least 12 triterpenoids have been isolated and reported from *L*. *camara*, with some of their analogues being less toxic on cells and others having CC_50s_ which go as low as <1 μM [26]. Like most or all drugs, lantadene A is toxic at higher doses, especially to livestock. Liver injury occurred after sheep were injected intravenously with lantadene A. A single dose of 1–3 mg/kg of the compound caused mild hepatocellular injury in sheep. Higher doses resulted in hepatic necrosis. It did not require metabolism in the alimentary tract for toxicity in sheep [25]. Reports of human toxicity by *L*. *camara* are rare. Most children with exploratory exposures to the plant remain asymptomatic. In the minority who develop mild effects, gastrointestinal irritation was most common. It is not known what substance produces these mild toxic effects in humans, but it does not appear that lantadene A or phototoxins contained therein are responsible [23]. Additional studies will therefore be needed to further investigate the anti-filarial effects of the other numerous compounds reported to be present in the plant by exploiting the structure–activity relationships to design better analogues that could be more active and non-toxic lead compounds for onchocerciasis treatment. The apparent low level of toxicity of lantadene A to humans could be supported by the current use of *L*. *camara* in traditional medicine of the present study population.

The IC_50s_ of 7.85 μg/mL for adult males, 10.38 μg/mL for adult females and 10 μg/mL for monkey kidney epithelial cells obtained for lantadene A were deemed encouraging suggesting that it could be a potential lead for the development of an onchocerciasis cure. Also the IC_50_ of 20.13 μg/mL of lantedene A for *L*. *loa* mf as compared to the IC_50_ of 10.84 μg/mL for *O*. *ochengi* mf is an indication that lantadene A is less active against *L*. *loa* and so could be somewhat safe to administer in areas of *Loa loa* co-endemicity. Further bioassay-guided fractional studies on the extracts are also recommended as we believe that we have not yet isolated all the main anti-*Onchocerca* principles from them. This study has thus, reported for the first time the anti-filarial activity of these medicinal plants and of lantadene A.
